# Effect of Cavity Disinfectants on Adhesion to Primary Teeth—A Systematic Review

**DOI:** 10.3390/ijms22094398

**Published:** 2021-04-22

**Authors:** Ana Coelho, Inês Amaro, Ana Apolónio, Anabela Paula, José Saraiva, Manuel Marques Ferreira, Carlos Miguel Marto, Eunice Carrilho

**Affiliations:** 1 Faculty of Medicine, Institute of Integrated Clinical Practice, University of Coimbra, 3000-075 Coimbra, Portugal; ines.amaros@hotmail.com (I.A.); anaclaudiia21@gmail.com (A.A.); anabelabppaula@sapo.pt (A.P.); ze-93@hotmail.com (J.S.); eunicecarrilho@gmail.com (E.C.); 2 Area of Environment Genetics and Oncobiology (CIMAGO), Faculty of Medicine, Coimbra Institute for Clinical and Biomedical Research (iCBR), University of Coimbra, 3000-548 Coimbra, Portugal; m.mferreira@netcabo.pt (M.M.F.); cmiguel.marto@uc.pt (C.M.M.); 3 Clinical Academic Center of Coimbra (CACC), 3004-561 Coimbra, Portugal; 4 Faculty of Medicine, Institute of Endodontics, University of Coimbra, 3000-075 Coimbra, Portugal; 5 Faculty of Medicine, Institute of Biophysics, University of Coimbra, 3004-548 Coimbra, Portugal; 6 Faculty of Medicine, Institute of Experimental Pathology, University of Coimbra, 3004-548 Coimbra, Portugal

**Keywords:** cavity disinfectants, primary teeth, adhesion, bond strength

## Abstract

Some authors have been proposing the use of cavity disinfectants in order to reduce, or even eliminate, the effect of the microorganisms present in a dental cavity before a restoration is placed. The aim of this study was to evaluate the effect of different cavity disinfectants on bond strength and clinical success of composite and glass ionomer restorations on primary teeth. The research was conducted using Cochrane Library, PubMed/MEDLINE, SCOPUS, and Web of Science for articles published up to February 2021. The search was performed according to the PICO strategy. The evaluation of the methodological quality of each in vitro study was assessed using the CONSORT checklist for reporting in vitro studies on dental materials. Sixteen in vitro studies and one in situ study fulfilled the inclusion criteria and were analyzed. Chlorhexidine was the most studied cavity disinfectant, and its use does not compromise dentin bonding. Sodium hypochlorite is a promising alternative, but more research on its use is required to clearly state that it can safely be used as a cavity disinfectant for primary teeth. Although other disinfectants were studied, there is a low-level evidence attesting their effects on adhesion, therefore their use should be avoided.

## 1. Introduction

Dental caries has a high prevalence worldwide, affecting more than 2.4 thousand million adults and 620 thousand children with primary teeth [1]. It can be defined as a multifactorial pathology arising from the interaction between dental structure and microbial biofilm, due to an imbalance between remineralization and demineralization, with the last one prevailing [2,3].

Although complete removal of the decayed and necrotic tissue is directly related to restorations’ clinical success, cariogenic bacteria can be pushed deep inside the dentinal tubules while removing the carious tissue and remain viable for a long time. In fact, the remaining of cariogenic bacteria in the cavity can be associated with the development of secondary caries [4,5].

According to Dalkilic et al. [6], fermenting microorganisms can remain viable for 139 days in a restored cavity. Moreover, bacteria present in the smear layer may remain viable and proliferate, allowing their metabolism products to reach and to cause inflammatory changes in the dental pulp. Bacteria penetration through restoration and teeth interface (microinfiltration) can also explain restorations’ failure [7,8,9].

As so, some authors have been proposing the use of cavity disinfectants in order to reduce, or even eliminate, the effect of the microorganisms present in a dental cavity before a restoration is placed [8,9,10].

Among the available disinfectants, chlorhexidine, sodium hypochlorite, and fluoridated solutions are the most used. Despite their benefits, their effect on adhesion to dentin, especially that of primary teeth, is still unknown [7,11,12].

Among the pediatric population, dental caries treatment is the most common procedure to be performed in a dental appointment [12]. Restorations’ success rate is associated with dentist’s experience and patient’s collaboration. However, one of the most common causes of failure is the development of secondary caries [13,14,15].

Thereby, the aim of this systematic review was to evaluate the effect of the application of different cavity disinfectants on bond strength and clinical success of composite and glass ionomer restorations on primary teeth.

## 2. Results

**Figure 1:**
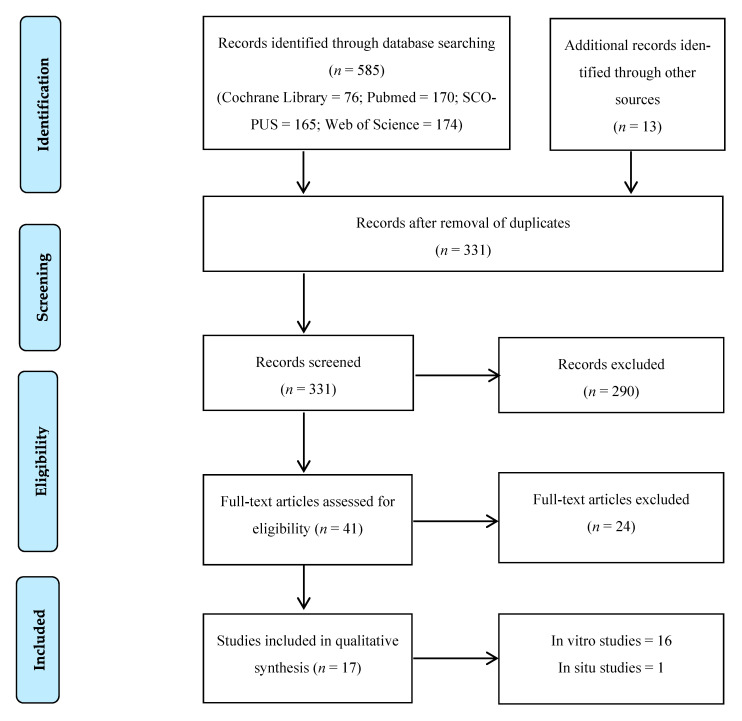
Flowchart of the data selection process.

Initial research on electronic databases resulted in 585 articles. After evaluating titles and abstracts, 41 articles were selected for full text analysis, and of those, 17 studies fulfilled the inclusion and exclusion criteria. The flowchart of the data selection process is detailed in Figure 1.

Sixteen in vitro studies [12,16,17,18,19,20,21,22,23,24,25,26,27,28,29,30] were included in this systematic review.

The earliest study was published in 2003 [12], and the most recent one in 2020 [29].

Most authors used primary molars [12,16,18,19,20,21,22,23,24,25,26,27,28,30], but Monghini et al. [17] evaluated canines, and Mohammadi et al. [29] used anterior teeth. Sample size varied from 2 [25] to 20 [28,31] teeth per group.

Even though all authors studied healthy dentin, Ersin et al. [18] additionally evaluated carious dentin, and Lenzi et al. [22,23] also evaluated demineralized dentin (artificially induced lesions).

After extraction, teeth were stored in thymol [12,18,28], chloramine [16,22,23,27,30], distilled water [16,20,22,23,24,25,27,30,32], saline solution [17,26], or sodium azide [17,21]. Ricci et al. [19] and Mohammadi [29] did not report data on the storage medium used after teeth extraction.

All authors used water to store the specimens after adhesive experiments and before bond strength evaluation.

All authors reported results on adhesion to composite resin. Only Ersin et al. [18] also reported results on adhesion to glass ionomer materials.

Most of the authors reported the use of 2% chlorhexidine [12,18,19,20,22,23,25,28,29] as a cavity disinfectant. A few studies reported results on the application of sodium hypochlorite [16,24,27], Er:YAG laser [17,21,26], KTP laser [25], ozone [25], doxycycline [29], ethylenediaminetetraacetic acid (EDTA) [29], propolis [25], and Aqua-prep™ (Bisco, Schaumburg, IL, USA) [26].

Except for Vieira et al. [12], all of the authors studying the effect of 2% chlorhexidine as a cavity disinfectant [18,19,20,22,23,25,28,29] reported positive results, allowing for maintenance or a statistically significant increase in bond strength values. These values ranged from 7.58 ± 3.18 MPa [25] to 66.45 ± 8.3 MPa [28] in resin specimens and from 7.1 ± 5.2 MPa to 14.4 ± 6.6 MPa [18] in caries-affected dentin in glass ionomer specimens. Vieira et al. [12] were the only authors applying chlorhexidine before etching the specimens with phosphoric acid.

The authors evaluating the effect of the application of sodium hypochlorite tested different concentrations, ranging from 2.5% [27] to 10% [16]. Regardless of the concentration, all authors [16,24,27] reported positive results, allowing for maintenance or even a statistically significant increase in bond strength values. These values ranged from 9.9 ± 0.2 MPa [16] to 18.45 ± 2.30 MPa [24].

The Er:YAG laser was evaluated by three studies [17,21,26]. Monghini et al. [17] reported statistically significant negative results when testing the laser with three different working parameters. However, Scatena et al. [21] did not find statistically significant differences regarding bond strength results for different focal distances (mm), and Yildiz et al. [26] even reported a statistically significant increase in bond strength values. The bond strength values of these studies ranged from 5.07 ± 2.62 MPa [21] to 20.57 ± 9.02 MPa [26].

Oznurhan et al. [25] assessed the use of a KTP laser as a cavity disinfectant and found no statistically significant differences when comparing its results to the ones of the control group (9.58 ± 2.92 and 6.38 ± 2.47 MPa, respectively).

Gaseous ozone and ozonated water [25] were also tested as cavity disinfectants. The authors reported a maintenance of the bond strength values when using gaseous ozone (5.84 ± 2.62 MPa vs. 6.38 ± 2.47 MPa for the control group) and a statistically significant increase of the bond strength values when using ozonated water (11.12 ± 2.41 MPa vs. 6.38 ± 2.47 MPa for the control group).

Aqua-prep™ [26], an aqueous solution of fluoride and hydroxyethyl methacrylate (HEMA), 2% Doxycycline [29], 17% EDTA [29], and 30% propolis [25] were all evaluated in only one study each, and no statistically significant differences were found between test and control groups.

Relevant information on each in vitro study is summarized in Table 1.

No clinical studies were identified, and only one in situ study regarding the use of a cavity disinfectant in primary teeth was evaluated. Ricci et al. [31] developed a split-mouth experimental protocol that included children aged between 8 and 11 years with at least two contralateral primary molars with small carious lesions. Chlorhexidine was used as a cavity disinfectant after enamel and dentin were etched with 35% phosphoric acid. The solution was removed with absorbent papers, and the cavities were restored with Prime & Bond NT^®^ (Dentsply, York, PA, USA) and Filtek™ Z250 (3M, Saint Paul, MN, USA). All the procedures were done under rubber dam, and the teeth were collected later, after exfoliation. The teeth were grouped according to the time of oral function after restoration: up to 30 days, 1 to 5 months, 10 to 12 months, and 18 to 20 months. A progressive decrease in bond strength values was reported for control and experimental groups as the time in oral function increased. However, a statistically significant decrease was reported sooner for the control group (it started after 1–5 months, while for the experimental group it started after 10–12 months). Also, significantly higher bond strength values were reported for the experimental group after 1–5 and 18–20 months.

### Quality Assessment

Methodological quality assessment outcomes are presented in Table 2. All studies presented accurate information regarding each item from 1 to 10. However, none of them provided results with confidence intervals. In addition, only two studies [26,29] reported study limitations and sources of potential bias (item 12).

## 3. Discussion

A cavity disinfectant must not only have a strong antimicrobial effect but also not compromise the adhesion of the restorative material to the dental substrates [7,32]. The majority of the studies on this topic reports results on permanent teeth, but the structural and mechanical properties of the primary teeth make it necessary to carry out experimental protocols testing this type of teeth [33,34]. Compared to permanent teeth, primary teeth have thinner enamel and dentin, are less mineralized due to their lower concentration of calcium and potassium ions, have a hybrid layer more prone to be degraded [35], and their dentin has a lower tubule density [18,36,37]. This may explain why bond strength values of composite materials in primary teeth are lower than those of permanent teeth [38].

Dental adhesion may be affected not only by the cavity disinfectant used but also by the dental substrate. In order to minimize its effect, it is recommended to perform adhesion tests in the superficial dentin of healthy teeth, ideally without restorations [39]. Deep dentin is mainly composed of dentinal tubules and a small percentage of intertubular dentin. Superficial dentin has a higher percentage of organic components (collagen) and of intertubular dentin and a lower number of dentinal tubules [40,41,42].

The differences between healthy and caries-affected dentin should also be underlined. The caries-affected dentin is more porous and softer due to its partial demineralization, which leads to a less effective adhesion [43,44,45]. In fact, some of the articles included in this systematic review evaluated the effect of a cavity disinfectant in healthy and affected dentin [18,22,23], and Lenzi et al. [22,23] reported significant lower bond strength values for the affected-dentin groups.

Besides dentin’s quality (superficial/deep dentin, permanent/primary teeth, healthy/carious dentin, amount of collagen and number, diameter, orientation, and size of dentinal tubules), moisture, contaminants, adhesive systems, solvents, and phosphoric acid/acidic primers are all factors affecting bond strength to dentin [46,47,48,49]. As so, the inclusion of at least one control group per study was mandatory for a study to be included in this systematic review.

All of the studies reported the use of a storage medium before the samples were submitted to the experimental protocol. The ISO/TS 11405/2015 (Dentistry–Testing of adhesion to tooth structure) [39] provides guidance for testing adhesion between dental substrates and restorative materials. This ISO/TS recommends the use of a 0.5% chloramine solution or of distilled water as a storage medium for the extracted teeth. If chloramine is chosen, it should be replaced by distilled water after one week. Despite these recommendations, some authors used other solutions, such as thymol [12,18,28]. The use of other solutions is not recommended by the ISO/TS 11405/2015, since it may affect dentin’s mechanical properties. In fact, Santana et al. [50] reported that the use of thymol as a storage medium led to impaired adhesion.

After the restorations were made, all authors stated that the samples were kept in water, which is exactly the recommendation of the ISO/TS 11405/2015 (ISO 3696:1987, grade 3) [51].

Almost all authors reported results on adhesion to molars, which is also in line with the recommendations of the ISO/TS 11405/201545. However, Monghini et al. [17] and Mohammadi et al. [29] used anterior teeth.

Most authors [12,18,19,20,22,23,25,28,29] evaluated the effect of chlorhexidine as a cavity disinfectant. Chlorhexidine has been widely used in dentistry, mainly because of its antimicrobial properties, including against *Streptococcus mutans*, and of its antiplaque effect [52,53,54,55]. Chlorhexidine is also well known for its ability to inhibit matrix metalloproteinases due to its strong collagenolytic activity, reducing the degradation of the hybrid layer [56,57], which may justify the positive results reported by almost all authors. Although only Ersin et al. [18] evaluated the effect of chlorhexidine on the adhesion to a glass ionomer material, the authors also reported positive results.

Similar results were previously reported for permanent teeth [58], which makes chlorhexidine the most consensual cavity disinfectant to be used in clinical practice. Not only adhesion to dentin is not only adequate after its use but, as stated by some authors [59,60], it can even be enhanced. As so, chlorhexidine presents as a safe and effective product to be used as a cavity disinfectant.

Sodium hypochlorite is commonly used as a cavity disinfectant due to its favorable properties: antibacterial action against aerobic bacteria, such as *S. mutans*, wettability, and deproteinization [61,62,63,64,65]. Although all authors studying the effect of the use of sodium hypochlorite as a cavity disinfectant in primary teeth reported positive results, only three articles [16,24,27] were identified. Since there are just a few studies reporting results on primary teeth and that the use of sodium hypochlorite as a cavity disinfectant in permanent teeth is still a matter of discussion [58], caution is required when choosing this product as a cavity disinfectant.

Initially presented as an alternative to the use of burs for cavity preparation, the Erbium:Ytrium (Er:YAG) laser was first introduced in 1989 by Hibst and Keller [66]. From then on, lasers have been used in numerous dentistry fields such as oral surgery, periodontics, endodontics, and prosthodontics [67]. However, similarly to what was reported for permanent teeth [58], there is no consensus regarding the use of lasers as cavity disinfectants, with some authors reporting an impairment of adhesion [17], and others reporting maintenance or even an enhancement of the bond strength values [21,26]. Moreover, even though some authors did not report secondary side effects [66,68,69], lasers may lead to overheating of the dental structures, which may induce pulp injuries, hydroxyapatite changes, and excessive dentin dehydration [17,70,71,72,73,74,75,76]. Given the results, the use of lasers as a cavity disinfection method should be avoided.

Both gaseous ozone and ozonated water have been recently introduced as alternatives to cavity disinfection due to their known antimicrobial and strong antioxidant properties. Polydorou et al. [77] reported that gaseous ozone eliminated 99.9% of the microorganisms in carious lesions in 20 s. In addition to its great antimicrobial activity (including against *S. mutans*), ozone also has antifungal and antiviral properties [78]. Authors analyzing the effect of either ozonated water or gaseous ozone on adhesion reported positive results [25], which may be justified by the opening of the dentinal tubules caused by oxygen [79,80,81,82,83]. Although there is limited information about the use of ozone as a cavity disinfectant in primary teeth, it looks like a promising alternative.

EDTA is an organic compound responsible for chelating calcium and potassium ions and for selective removal of hydroxyapatite crystals, which allows for the maintenance of the collagen matrix [84,85]. It is widely used in endodontics to improve shaping of the entire root canal system and to dissolve the inorganic components of the smear layer [86]. Although the reported results were positive (no differences on bond strength values after using it as a cavity disinfectant), only one study [29] evaluated it. A few articles on permanent teeth [58] also showed that EDTA presents as a promising alternative, but there is a clear need for further research.

Aqua-prep^TM^ [26], 2% doxycycline [29], and 30% propolis [25] were all evaluated by studies included in this review, and the reported results were positive, but only one article was included for each product. Given the limited scientific evidence associated with these products (even in permanent teeth [58]), their use as cavity disinfectants should be avoided.

The limitations of this systematic review mainly reflect the shortcomings of the included articles. No clinical studies on the topic were identified, and such studies are essential to analyze the effects of the different cavity disinfectants when applied in the oral cavity. In addition, there is no information on the best application time and on the durability of bond interfaces over time. Also, there are several studies reporting results on different adhesive systems (total etch, self-etch, universal) but given the different methods applied, it is impossible to draw conclusions regarding this matter.

Further studies with standardized protocols should be developed to allow solid conclusions and recommendations concerning this issue. The effect of the incorporation of cavity disinfectants into adhesive systems must also be evaluated, since it may reduce clinical steps, which is of great importance in pediatric dentistry.

## 4. Materials and Methods

The present systematic review was registered on the International Prospective Register of Systematic Reviews (PROSPERO) platform (ID CRD42020199614) and followed the PRISMA protocol (Preferred Reporting Items for Systematic Reviews and Meta-Analyses Protocols) [87].

The research questions were developed according to the PICO (Population, Intervention, Comparison, Outcome) methodology, as described in Table 3.

The inclusion and exclusion criteria are presented in Table 4.

An electronic research was conducted in Cochrane Library (www.cochranelibrary.com), PubMed/MEDLINE (pubmed.ncbi.nlm.nih.gov), SCOPUS (www.scopus.com), and Web of Science (webofknowledge.com). The research keys used in each database can be found in Table 5.

The search was limited to articles published until 14 February 2021, with no restrictions on region, language, or year of publication. A manual search for other references in reviews and in the included articles was performed.

Duplicate articles were removed with Endnote 20 (Clarivate™, Boston, MA, USA). Two independent reviewers analyzed titles, abstracts, and full texts, and a third one’s opinion was obtained when necessary.

Selected articles were read by the same two independent authors, who collected the following data on the in vitro studies: authors and year of publication, number of elements per group (n), materials used (cavity disinfectant, type of adhesive system, and restorative material), storage, and bond strength results.

Regarding the clinical/in situ studies, the following data were acquired: authors and year of publication, type of teeth, number and ages of children per group (n), materials used (cavity disinfectant, type of adhesive system, and restorative material), and results (pigmentation, marginal gaps, or existence of carious lesions).

### Quality Assessment

The evaluation of the methodological quality of each in vitro study was assessed using the modified Consolidated Standards of Reporting Trials (CONSORT) checklist [88] for reporting in vitro studies on dental materials. When applying this checklist, items 5 to 9 could not be evaluated, since these are designed to evaluate sample standardization. Two authors assessed the risk of bias independently, and any disagreement was solved by consensus.

## 5. Conclusions

Chlorhexidine is the most studied cavity disinfectant, and according to the results, its use does not compromise adhesion to primary dentin. Sodium hypochlorite is a promising alternative, but more research on its effects on adhesion is required to clearly state that it can be safely used as a cavity disinfectant for primary teeth. Although other disinfectants were studied, there is a low-level evidence attesting their effects on adhesion; therefore, their use should be avoided.

There is a clear need for researchers to conduct well-designed in vitro and clinical studies so more options can be identified, and the long-term effect on adhesion can be evaluated.

## Figures and Tables

**Table 1:** Characteristics of the in vitro studies included in the systematic review.

Authors, Year	Groups (n)	Teeth	Storage	Materials	Results (MPa)
Vieira et al., 2003 [12]	G_1_—37% phosphoric acid + adhesive (10) + resin G_2_—2% CHX + 37% phosphoric acid + adhesive (10) + resin	Molars	0.1% Thymol	Adhesive: 3M Single Bond (3M, USA) Resin: Filtek^TM^ Z250 (3M, USA)	G_1_: 19.88 ± 1.04 G_2_: 17.99 ± 1.15 G_1_*/G_2_
Correr et al., 2004 [16]	G_1_—35% phosphoric acid + adhesive 1 (15) G_2_—35% phosphoric acid + 10% NaOCl + adhesive 1 (15) G_3_—37% phosphoric acid + adhesive 2 (15) G_4_—37% phosphoric acid + 10% NaOCl + adhesive 2 (15) G_5_–Adhesive 3 (15) G_6_—10% NaOCl + adhesive 3 (15) + resin	Molars	0.5% Chloramine	Adhesive: 1–3M Single Bond; 2–Prime & Bond 2.1^®^ (Dentsply, Brazil); 3–Clearfill^TM^ SE Bond (Kuraray, Houston, TX, USA) Resin: Filtek^TM^ Z250 (3M, USA)	G_1_: 15.8 ± 1.9 G_2_: 14.6 ± 1.3 G_3_: 10.2 ± 0.7 G_4_: 9.9 ± 0.2 G_5_: 13.3 ± 1.2 G_6_: 10.7 ± 1.0 G_1_*/G_3_
Monghini et al., 2004 [17]	G_1_—None (12) G_2_—Laser Er;YAG 60 mJ/2 Hz (12) G_3_—Laser Er;YAG 80 mJ/2 Hz (12) G_4_—Laser Er;YAG 100 mJ/2 Hz (12) + 35% phosphoric acid + adhesive + resin	Canines	0.9% Saline solution with 0.4% sodium azide	Adhesive: 3M Single Bond Laser: Kavo Key Laser 2 (Kavo Dental, Germany) Resin: Filtek^TM^ Z250	G_1_:17.89 ± 4.75 G_2_:12.34 ± 4.85 G_3_:10.30 ± 3.67 G_4_:10.41 ± 4.20 G_1_*/G_2_;G_3_;G_4_
Ersin et al., 2009 [18]	G_1_—25% polyacrlylic acid + 2% CHX + GIC 1 (sound dentin) (3) G_2_—25% polyacrlylic acid + 2% CHX + GIC 1 (carious dentin) (3) G_3_—25% polyacrlylic acid + GIC 1 (sound dentin) (3) G_4_—25% polyacrlylic acid + GIC 1 (carious dentin) (3) G_5_—2% CHX + GIC 2 (sound dentin) (3) G_6_—2% CHX + GIC 2 (carious dentin) (3) G_7_—GIC 2 (sound dentin) (3) G_8_—GIC 2 (carious dentin) (3) G_9_—37% phosphoric acid + 2% CHX + adhesive + resin (sound dentin) (3) G_10_–37% phosphoric acid + 2% CHX + adhesive + resin (carious dentin) (3) G_11_—37% phosphoric acid + adhesive + resin (sound dentin) (3) G_12_—37% phosphoric acid + adhesive + resin (carious dentin) (3)	Molars	0.1% Thymol	Adhesive: Prime & Bond^®^; GIC: 1–Ketac^TM^ Molar (3M, Germany); 2–Vitremer^TM^ (3M, USA) Resin–Surefil^TM^ (Dentsply, USA)	G_1_: 8.7 ± 4.3 G_2_: 7.1 ± 5.2 G_3_: 9.2 ± 5.2 G_4_: 10.3 ± 6.6 G_5_: 12.4 ± 5.7 G_6_: 14.4 ± 6.6 G_7_: 11.2 ± 4.8 G_8_: 13.8 ± 4.9 G_9_: 22.9 ± 6.9 G_10_: 23.2 ± 6.2 G_11_: 20.2 ± 5.8 G_12_: 22.1 ± 6.2 G_9_*/G_1_;G_2_;G_3_;G_4_;G_5_;G_6_;G_7_;G_8_ G_10_*/G_1_;G_2_;G_3_;G_4_;G_5_;G_6_;G_7_;G_8_ G_11_*/G_1_;G_2_;G_3_;G_4_;G_5_;G_6_;G_7_;G_8_ G_12_*/G_1_;G_2_;G_3_;G_4_;G_5_;G_6_;G_7_;G_8_
Ricci et al., 2010 [19]	35% phosphoric acid + G_1_—2% CHX + adhesive 1 (4) G_2_—deionized water + adhesive 1 (4) G_3_—2% CHX + adhesive 2 (4) G_4_—deionized water + adhesive 2 (4) G_5_—2% CHX + adhesive 3 (4) G_6_—deionized water + adhesive 3 (4) + resin	Molars	NA	Adhesive: 1–Adper^TM^ Single Bond (3M, USA); 2–Prime & Bond NT^®^ (Dentsply, USA); 3–Excite^®^ DSC (Ivoclar, Liechtenstein) Resin: Filtek^TM^ Z250	G_1_: 47.4 ± 9.5 G_2_: 41.4 ± 11.9 G_3_: 48.0 ± 9.8 G_4_: 40.8 ± 13.4 G_5_: 45.2 ± 9.2 G_6_: 43.4 ± 12.0 G_1_*/G_2_; G_3_*/G_4_
Leitune et al., 2011 [20]	37% phosphoric acid + G_1_—Adhesive (24 h) (10) G_2_—Adhesive (6 months) (10) G_3_—2% CHX + Adhesive (24 h) (10) G_4_—2% CHX + Adhesive (6 months) (10)	Molars	Distilled water	Adhesive: Adper^TM^ Scotchbond^TM^ Multi Purpose (3M, USA) Resin: Filtek^TM^ Z250	G_1_: 22.37 ± 3.69 G_2_: 19.93 ± 2.05 G_3_: 22.30 ± 3.66 G_4_: 24.48 ± 2.24 G_2_*/G_4_
Scatena et al., 2011 [21]	G_1_–None (10) G_2_—Laser Er:YAG (80 mJ, 11 mm) (10) G_3_—Laser Er:YAG (80 mJ, 12 mm) (10) G_4_—Laser Er:YAG (80 mJ, 16 mm) (10) G_5_—Laser Er:YAG (80 mJ, 17 mm) (10) G_6_—Laser Er:YAG (80 mJ, 20 mm) (10) + 37% phosphoric acid + adhesive + resin	Molars	0.4% Sodium azide	Laser: Kavo Key Laser 2 Adhesive: 3M Single Bond Resin: Filtek^TM^ Z250	G_1_: 7.32 ± 3.83 G_2_: 5.07 ± 2.62 G_3_: 6.49 ± 1.64 G_4_: 7.71 ± 0.66 G_5_: 7.33 ± 0.02 G_6_: 9.65 ± 2.41 G_2_*/G_4_;G_6_
Manfro et al., 2012 [30]	37% phosphoric acid + G_1_—water + adhesive (7) G_2_—water + adhesive (12 months) (7) G_3_—0.5% CHX + adhesive (7) G_4_—0.5% CHX + adhesive (12 months) (7) G_5_—2% CHX + adhesive (7) G_6_—2% CHX + adhesive (12 months) (7) + resin	Molars	0.5% Chloramine	Adhesive: 3M Single Bond Resin: Filtek^TM^ Z250	G_1_: 50.8 ± 12.8 G_2_: 20.4 ± 3.7 G_3_: 49.3 ± 2.6 G_4_: 32.3 ± 7.9 G_5_: 44.0 ± 8.7 G_6_: 34.6 ± 5.1 G_1_*/G_2_; G_2_*/G_4_;G_6_; G_3_*/G_4_; G_5_*/G_6_
Lenzi et al., 2012 [22]	35% phosphoric acid + G_1_—distilled water + adhesive (sound dentin) (5) G_2_—2% CHX + adhesive (sound dentin) (5) G_3_—distilled water + adhesive (artificial caries) (5) G_4_—2% CHX + adhesive (artificial caries) (5)	Molars	0.5% Chloramine	Adhesive: Adper^TM^ Single Bond 2 Resin: Filtek^TM^ Z250	G_1_: 30.8 ± 2.2 G_2_: 32.8 ± 3.8 G_3_: 24.5 ± 3.8 G_4_: 25.6 ± 3.6 G_1_*/G_3_;G_4_; G_2_*/G_3_;G_4_
Aras et al., 2013 [24]	G_1_—37% phosphoric acid (10) G_2_—37% phosphoric acid + 5% NaOCl (10) G_3_—5% NaOCl + 37% phosphoric acid (10) + adhesive + resin	Molars	Distilled water	Adhesive: Gluma^®^ Confort Bond (Herause-Kulzer, Germany) Resin: Charisma^®^ (Herause-Kulzer, Germany)	G_1_: 14.51 ± 2.89 G_2_: 18.45 ± 2.30 G_3_: 17.06 ± 2.99 G_1_*/G_2_
Lenzi et al., 2014 [23]	35% phosphoric acid + G_1_—distilled water + adhesive (sound dentin) (5) G_2_—distilled water + adhesive (sound dentin) (6 months) (5) G_3_—2% CHX (without rinsing) + adhesive (sound dentin) (5) G_4_—2% CHX (without rinsing) + adhesive (sound dentin) (6 months) (5) G_5_—distilled water + adhesive (artificial lesion) (5) G_6_—distilled water + adhesive (artificial lesion) (6 months) (5) G_7_—2% CHX (without rinsing) + adhesive (artificial lesion) (5) G_8_—2% CHX (without rinsing) + adhesive (artificial lesion) (6 months) (5)	Molars	Distilled water	Adhesive: Adper^TM^ Single Bond Resin: Filtek^TM^ Z250	G_1_: 30.7 ± 2.2 G_2_: 25.9 ± 5.7 G_3_: 32.8 ± 3.8 G_4_: 31.3 ± 2.6 G^5^: 26.2 ± 5.4 G_6_: 20.0 ± 3.9 G_7_: 28.3 ± 3.4 G_8_: 26.9 ± 5.9 G_1_*/G_5_;G_7_; G_2_*/G_6_;G_8_ G_3_*/G_5_;G_7_ G_4_*/G_6_;G_8_
Oznurhan et al., 2015 [25]	G_1_—2% CHX (2) G_2_—30% propolis (2) G_3_—Gaseous ozone (2) G_4_—Ozonated water (2) G_5_—Laser KTP (2) G_6_—None (2) + adhesive + resin	Molars	Distilled water	Adhesive: Adper^TM^ Prime & Bond NT^®^ Resin: Tetric^®^ N-Ceram (Ivoclar Vivadent, Liechenstein) Laser: Smartlite D (Deka, Italy)	G_1_: 7.58 ± 3.18 G_2_: 7.42 ± 2.28 G_3_: 5.84 ± 2.62 G_4_: 11.12 ± 2.41 G_5_: 9.58 ± 2.92 G_6_: 6.38 ± 2.47 G_3_*/G_5_; G_4_*/G_1_/G_2_/G_3_/G_6_
Yildiz et al., 2015 [26]	G_1_—37% phosphoric acid (3) G_2_—37% phosphoric acid + Aqua-Prep™ (without rinsing) (3) G_3_—Laser Er:YAG (10 Hz, 8 mm) (3) + adhesive + resin	Molars	Saline solution	Adhesive: Adper^TM^ Single Bond 2 Resin: Filtek^TM^ Z250 Laser: Fidelis Plus III (Fotona, Slovenia) Aqua-Prep^TM^ (Bisco, USA)	G_1_: 14.28 ± 5.22 G_2_: 18.35 ± 7.94 G_3_: 20.57 ± 9.02 G_1_*/G_3_
Bahrololoomi et al., 2017 [27]	35% phosphoric acid + G_1_–none (14) G_2_–2.5% NaOCl (14) G_3_–5.25% NaOCl (14) + adhesive + resin	Molars	0.5% Chloramine	Adhesive: One-Step^®^ Plus (Bisco, USA) Resin: AELITE (Bisco, USA)	G_1_: 13.56 ± 3.36 G_2_: 13.53 ± 3.64 G_3_: 14.36 ± 3.64
Ebrahimi et al., 2018 [28]	G_1_—37% phosphoric acid + adhesive 1 (20) G_2_—37% phosphoric acid + adhesive 1 (3 months) (20) G_3_—37% phosphoric acid + adhesive 1 + 2% CHX (without rinsing) (20) G_4_—37% phosphoric acid + adhesive 1 + 2% CHX (without rinsing) (3 months) (20) G_5_—Adhesive 2 (20) G_6_—Adhesive 2 (3 months) (20) G_7_—Adhesive 2 (Primer) + 2% CHX (without rinsing) + adhesive 2 (bond) (20) G_8_—Adhesive 2 (primer) + 2% CHX (without rinsing) + adhesive 2 (bond) (3months) (20)	Molars	0.1% Thymol + water	Adhesive: 1–Adper^TM^ Single Bond 2–Clearfil^TM^ SE Bond Resin: Filtek^TM^ Z250	G_1_: 25.43 ± 12.94 G_2_: 39.96 ± 21.75 G_3_: 66.45 ± 8.3 G_4_: 39.02 ± 23.29 G_5_: 47.83 ± 19.83 G_6_: 53.36 ± 18.05 G_7_: 46.25 ± 9.34 G_8_: 56.4 ± 22.18 G_1_*/G_3_
Mohammadi et al., 2020 [29]	37% phosphoric acid + G_1_–PBS (15) G_2_—2% CHX (without rinsing) (15) G_3_—2% Doxycycline (without rinsing) (15) G_4_—17% EDTA (15) + adhesive	Anterior teeth	-	Adhesive: Adper^TM^ Single Bond 2 Resin: Filtek^TM^ Z250	G_1_: 6.20 ± 2.11 G_2_: 5.60 ± 2.69 G_3_: 8.82 ± 3.29 G_4_: 7.50 ± 3.94 G_2_*/G_3_

CHX–Chlorhexidine; EDTA–Ethylenediaminetetraacetic Acid; GIC–Glass Ionomer Cement; NaOCl–Sodium hypochlorite; *–Statistically significant difference (*p* < 0.05).

**Table 2:** Modified CONSORT checklist for reporting in vitro studies of dental materials.

Studies	Item									
	1 Abstract	2a Introduction (Background)	2b Introduction (Objectives)	3 Methods (Intervention)	4 Methods (Outcomes)	10 Methods (Statistical Methods)	11 Results (Outcomes and Estimation)	12 Discussion (Limitations)	13 Other Information (Funding)	14 Other Information (Protocol)
Vieira et al., 2003 [12]	Yes	Yes	Yes	Yes	Yes	Yes	Yes ^a^	No	No	No
Correr et al., 2004 [16]	Yes	Yes	Yes	Yes	Yes	Yes	Yes ^a^	No	No	No
Monghini et al., 2004 [17]	Yes	Yes	Yes	Yes	Yes	Yes	Yes ^a^	No	No	No
Ersin et al., 2009 [18]	Yes	Yes	Yes	Yes	Yes	Yes	Yes ^a^	No	No	No
Ricci et al., 2010 [19]	Yes	Yes	Yes	Yes	Yes	Yes	Yes ^a^	No	Yes	No
Leitune et al., 2011 [20]	Yes	Yes	Yes	Yes	Yes	Yes	Yes ^a^	No	No	No
Scatena et al., 2011 [21]	Yes	Yes	Yes	Yes	Yes	Yes	Yes ^a^	No	No	No
Manfro et al., 2012 [30]	Yes	Yes	Yes	Yes	Yes	Yes	Yes ^a^	No	No	No
Lenzi et al., 2012 [22]	Yes	Yes	Yes	Yes	Yes	Yes	Yes ^a^	No	Yes	No
Aras et al., 2013 [24]	Yes	Yes	Yes	Yes	Yes	Yes	Yes ^a^	No	Yes	No
Lenzi et al., 2014 [23]	Yes	Yes	Yes	Yes	Yes	Yes	Yes ^a^	No	Yes	No
Oznurhan et al., 2015 [25]	Yes	Yes	Yes	Yes	Yes	Yes	Yes ^a^	No	Yes	No
Yildiz et al., 2015 [26]	Yes	Yes	Yes	Yes	Yes	Yes	Yes ^a^	Yes	Yes	No
Bahrololoomi et al., 2017 [27]	Yes	Yes	Yes	Yes	Yes	Yes	Yes ^a^	No	Yes	No
Ebrahimi et al., 2018 [28]	Yes	Yes	Yes	Yes	Yes	Yes	Yes ^a^	No	Yes	No
Mohammadi et al., 2020 [29]	Yes	Yes	Yes	Yes	Yes	Yes	Yes ^a^	Yes	Yes	No

^a^ No confidence interval.

**Table 3:** Problem, Intervention, Comparison, Outcome (PICO) strategy.

Parameter	In Vitro Studies	Clinical/In Situ Studies
P (Population)	Primary teeth / dentin discs	Children in need of a restoration
I (Intervention)	Restoration with prior application of a cavity disinfectant	
C (Comparison)	Conventional restoration	
O (Outcome)	Effect of cavity disinfection on dentin bond strength	Effect of cavity disinfection on clinical success

**Table 4:** Inclusion and Exclusion Criteria.

Inclusion Criteria	Primary teeth evaluation
	Bond strength/clinical success evaluation
	Existence of a control group
	Evaluation of commercially available adhesive systems and composite resins or glass ionomer
	Application of only one cavity disinfectant per experimental group
	Report of results as mean and standard deviation
Exclusion Criteria	Permanent teeth evaluation
	Evaluation of teeth with endodontic treatment
	Evaluation of adhesion of cements, posts, sealants, or brackets
	Use of experimental adhesive systems or of mixtures of adhesives with disinfectants
	Revisions, animal or cell studies, letters, abstracts, comments, and clinical cases

**Table 5:** Search keys used in the different databases.

Database	Search keys
Cochrane Library	#1 MeSH descriptor: [Dentin] explode all trees
	#2 dentin
	#3 cavity
	#4 MeSH descriptor: [Disinfection] explode all trees
	#5 disinfect*
	#6 antibacteria*
	#7 MeSH descriptor: [Anti-Bacterial Agents] explode all trees
	#8 chlorhexidine
	#9 MeSH descriptor: [Chlorhexidine] explode all trees
	#10 “sodium hypochlorite”
	#11 MeSH descriptor: [Sodium Hypochlorite] explode all trees
	#12 laser
	#13 MeSH descriptor: [Lasers] explode all trees
	#14 ozone
	#15 MeSH descriptor: [Ozone] explode all trees
	#16 “aloe vera”
	#17 MeSH descriptor: [Aloe] explode all trees
	#18 ethanol
	#19 MeSH descriptor: [Ethanol] explode all trees
	#20 EDTA
	#21 MeSH descriptor: [Edetic Acid] explode all trees
	#22 “green tea”
	#23 EGCG
	#24 “bond strength”
	#25 adhesion
	#26 adhesive
	#27 MeSH descriptor: [Dental Cements] explode all trees
	#28 primary
	#29 deciduous
	#30 MeSH descriptor: [Tooth, Deciduous] explode all trees
	#31 temporary
	#32 #1 OR #2 OR #3
	#33 #4 OR #5 OR #6 OR #7 OR #8 OR #9 OR #10 OR #11 OR #12 OR #13 OR #14 OR #15 OR #16 OR #17 OR #18 OR #19 OR #20 OR #21 OR #22 OR #23
	#34 #24 OR #25 OR #26 OR #27
	#35 #28 OR #29 OR #30 OR #31
	#36 #32 AND #33 AND #34 AND #35
PubMed	(dentin[MeSH Terms] OR dentin OR cavity) AND (disinfection[MeSH Terms] OR disinfect* OR antibacteria* OR agents, antibacterial[MeSH Terms] OR chlorhexidine[MeSH Terms] OR chlorhexidine OR “sodium hypochlorite” OR sodium hypochlorite[MeSH Terms] OR laser OR lasers[MeSH Terms] OR ozone OR ozone[MeSH Terms] OR “aloe vera” OR aloe[MeSH Terms] OR ethanol OR ethanol[MeSH Terms] OR EDTA OR Edetic acid[MeSH Terms] OR “green tea” OR EGCG) AND (“bond strength” OR adhesion OR adhesive OR adhesives[MeSH Terms]) AND (deciduous tooth[MeSH Terms] OR deciduous OR primary OR temporary)
SCOPUS	TITLE-ABS-KEY (dentin OR cavity) AND TITLE-ABS-KEY (disinfect* OR antibacterial* OR chlorhexidine OR “sodium hypochlorite” OR laser OR ozone OR “aloe vera” OR ethanol OR EDTA OR “green tea” OR EGCG) AND TITLE-ABS-KEY (“bond strength” OR adhesion OR adhesive) AND TITLE-ABS-KEY (primary OR deciduous OR temporary)
Web of Science	TS= ((dentin[MeSH Terms] OR dentin OR cavity) AND (disinfect* OR antibacteria* OR chlorhexidine OR “sodium hypochlorite” OR laser OR ozone OR “aloe vera” OR ethanol OR EDTA OR “green tea” OR EGCG) AND (“bond strength” OR adhesion or adhesive) AND (primary OR deciduous OR temporary))
